# Retraction: The Human Homolog of Drosophila Headcase Acts as a Tumor Suppressor through Its Blocking Effect on the Cell Cycle in Hepatocellular Carcinoma

**DOI:** 10.1371/journal.pone.0215040

**Published:** 2019-04-02

**Authors:** Jun Wang, Li Gong, Shao-Jun Zhu, Qiao Zhu, Li Yao, Xiu-Juan Han, Jia-Rui Zhang, Yan-Hong Li, Wei Zhang, The PLOS ONE Editors

Following publication of this article [1], concerns were raised regarding possible duplications in images presented in Figs 4 and 5:

• In Fig 4B, the SNC and HECA exp panels for Huh-7 are photos of the same membrane taken under different microscope views.

• In Fig 4B, the siRNA, SNC, and mock panels for MHCC-97H are photos of the same membrane taken under different microscope views.

• In Fig 5B, the siRNA and SNC flow cytometry panels for MHCC-97H appear similar.

The authors have verified that there were errors in the preparation of Fig 4 as stated above, and replacement figure panels were provided. The authors stand by the flow cytometry dot plots in Fig 5B as originating from independent samples, stating that the similarity may be ascribed to the similar effects of siRNA and SNC to the MHCC-97 cell line.

The flow cytometry experiments investigating cell cycle and apoptosis were performed by the Central Lab of Tangdu Hospital as a paid service, which was not declared in the published article. The authors have stated that raw .fcs files cannot be provided by the Central Lab, which only issued PDF format data for flow cytometry experiments. The exported PDFs of the representative dot plots were provided by the authors, along with the primary data underlying all other figures in the article.

When initially alerted to the errors in the article, the authors raised to the attention of the *PLOS ONE* Editors that a discrepancy in the data presented in Fig 5B was not adequately addressed prior to publication of the article. Specifically, over-expression of HECA can increase the apoptosis rate in the HepG2 cell line, but not in the Huh-7 or MHCC-97H cell lines. The authors requested retraction and have carried out follow-up experiments using stable overexpression of HECA by lentiviral infection in HepG2 cells to provide support for the conclusions of the original article.

The *PLOS ONE* Editors have evaluated the information and files available and have consulted with a member of the editorial board. It was concluded that the new experimental data provided by the authors improve the study. However, the concerns raised regarding the flow cytometry panels in Fig 5B remain unresolved and the raw flow cytometry data files for the follow-up experiments are also unavailable. In light of these concerns, the authors and the *PLOS ONE* Editors retract the article.

The authors agree with this retraction and apologize for the issues noted above.

JW, LG, S-JZ, QZ, LY, X-JH, J-RZ, Y-HL, WZ agreed with the retraction.
